# Genomic distance entrained clustering and regression modelling highlights interacting genomic regions contributing to proliferation in breast cancer

**DOI:** 10.1186/1752-0509-4-127

**Published:** 2010-09-08

**Authors:** Tim J Dexter, David Sims, Costas Mitsopoulos, Alan Mackay, Anita Grigoriadis, Amar S Ahmad, Marketa Zvelebil

**Affiliations:** 1 Breakthrough Breast Cancer Research Centre, Institute of Cancer Research, Chester Beatty Laboratories, 237 Fulham Road, London, SW3 6JB, UK

## Abstract

**Background:**

Genomic copy number changes and regional alterations in epigenetic states have been linked to grade in breast cancer. However, the relative contribution of specific alterations to the pathology of different breast cancer subtypes remains unclear. The heterogeneity and interplay of genomic and epigenetic variations means that large datasets and statistical data mining methods are required to uncover recurrent patterns that are likely to be important in cancer progression.

**Results:**

We employed ridge regression to model the relationship between regional changes in gene expression and proliferation. Regional features were extracted from tumour gene expression data using a novel clustering method, called genomic distance entrained agglomerative (GDEC) clustering. Using gene expression data in this way provides a simple means of integrating the phenotypic effects of both copy number aberrations and alterations in chromatin state. We show that regional metagenes derived from GDEC clustering are representative of recurrent regions of epigenetic regulation or copy number aberrations in breast cancer. Furthermore, detected patterns of genomic alterations are conserved across independent oestrogen receptor positive breast cancer datasets. Sequential competitive metagene selection was used to reveal the relative importance of genomic regions in predicting proliferation rate. The predictive model suggested additive interactions between the most informative regions such as 8p22-12 and 8q13-22.

**Conclusions:**

Data-mining of large-scale microarray gene expression datasets can reveal regional clusters of co-ordinate gene expression, independent of cause. By correlating these clusters with tumour proliferation we have identified a number of genomic regions that act together to promote proliferation in ER+ breast cancer. Identification of such regions should enable prioritisation of genomic regions for combinatorial functional studies to pinpoint the key genes and interactions contributing to tumourigenicity.

## Background

The field of breast cancer research was amongst the first to adopt genomic profiling tools such as competitive genomic hybridisation (aCGH) and DNA methylation analysis in order to investigate the molecular basis of disease progression. Studies using aCGH to examine DNA copy number changes in breast tumours have demonstrated that the copy number aberrations (CNAs) are not random, but are more prevalent in particular chromosomal locations [1-4]. Indeed, it has become evident that patterns of genomic rearrangements differ between disease subtypes, and may be of prognostic significance [1-4]. It is clear from these studies that particular genomic copy number aberrations are associated with tumour grade. Furthermore, local DNA copy number changes have been shown to cause gene expression changes such that a majority of the genes in gained or amplified regions exhibit increased expression [5].

Similarly, regional epigenetic changes involving DNA methylation and chromatin structure which lead to or stabilize altered gene expression have been shown to be involved in breast cancer [6]. The interplay of alterations in DNA copy number and epigenetic states is complex, and to understand the full picture data from multiple sources needs to be integrated. Since both copy number and epigenetic alterations result in changes in gene expression patterns, analysis of microarray gene expression data in the context of specific genomic regions is an efficient means of integrating the effects of genomic changes in cancer.

Oestrogen receptor positive (ER+) breast cancer represents the most prevalent breast cancer subtype, and although several anti-oestrogen therapies are available to treat hormone dependent disease, resistance to therapy is common and the full molecular basis of the disease is not fully understood. In this study we have assembled data from ER+ tumours within five published large-scale microarray gene expression datasets and developed a computational analysis approach to score the contributions of genomic regions with altered gene expression to proliferation and hence grade.

Previous analysis of gene expression profiles from ER+ breast tumours has implicated a set of highly correlated genes involved in cell proliferation as a key prognostic feature [7]. This proliferation signature is highly enriched with genes known to be cell-cycle regulated and therefore provides an array-based mitotic index [7-9]. When correlates of histological grade were sought in gene expression profiles, most of the genes selected were those previously found in the proliferation signature [10,11]. Moreover, it has been demonstrated that the array-based "Genomic Grade Index" was, at least for ER+ breast cancer, more accurate than histological grade in predicting clinical outcome [12]. We explore the relationship of the proliferation signature to genomic regions that display marked covariant gene expression across a large number of tumours.

Patterns of gene expression that are associated with particular aspects of sample phenotype are often referred to as "signatures". This term has been used quite broadly both for clusters of co-regulated and thus correlated genes such as in the proliferation signature [7-9], but also for more complex expression profiles that involve a number of loosely, or even inversely, correlated gene clusters. Like others [13,14] we have adopted the more operationally defined and analytically useful metagene approach, in which clusters of correlated genes are replaced by statistical summaries of them; here we use cluster centroids (mean vectors). The metagene approach sacrifices detail at the individual gene level in order to gain statistical robustness, generalisability and the necessary dimension reduction to enable higher-level analysis.

The analysis of gene expression data from ER+ breast cancer that we present here involves a number of stages. Firstly, we describe a novel clustering algorithm (GDEC) that uses genomic distance together with expression data to reveal regional patterns of co-ordinate gene expression. We show that many, but importantly not all, of these regional clusters reflect common CNAs in this type of cancer. We derive metagenes as cluster centroids and we refer to metagenes derived from GDEC clusters as regional metagenes (RMGs). We use regression analysis with the RMGs to identify the most important regions for the prediction of proliferation as defined by the proliferation metagene.

## Results and Discussion

### Genomic distance entrained clustering

We have developed a novel clustering method, called Genomic Distance (GDEC) Entrained Clustering, to identify genomic regions where gene expression is co-ordinately altered. The algorithm reduces the correlation distance between genes in the same chromosomal neighbourhood in a genomic distance and correlation dependent manner. This type of data clustering is generically known as clustering with side-information or clustering with soft constraints and is more typically used in geographical applications [15]. Details of the algorithm and the parameters used are provided in the methods section.

**Figure 1:**
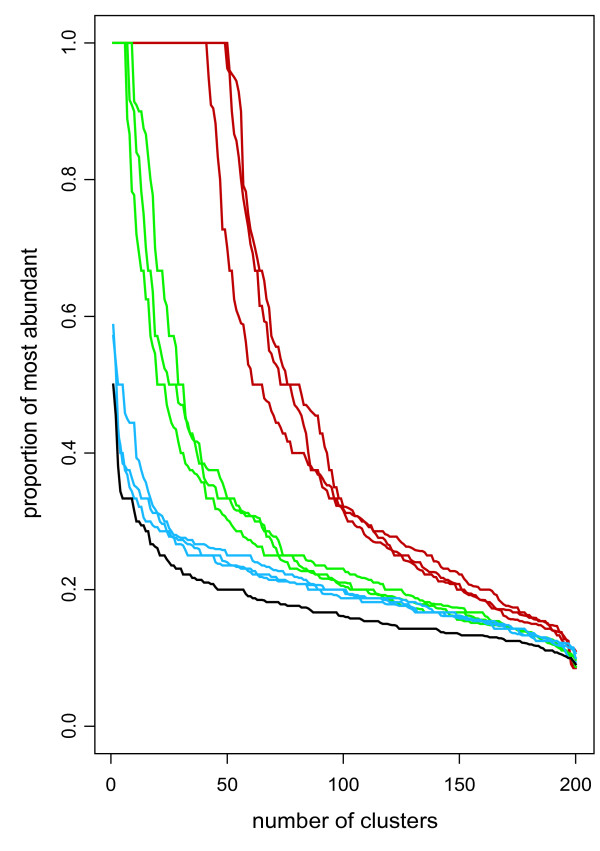
**Enrichment of clusters with genes from the same chromosome**. The dendrograms resulting from standard flexible beta and GDEC clustering were cut at 200 clusters. The chromosomal locations of the genes in each cluster were tabulated, and for each cluster the number of genes from the most abundantly represented chromosome was scored as a fraction of the number of genes in the cluster. These proportion scores are plotted in ranked order from left to right for each of the three largest datasets. The red lines indicate GDEC clustering, the green lines standard flexible beta clustering and the blue lines GDEC clustering after destruction of correlation structure by permutation. The single black line indicates the profile obtained by randomly assigning genes to clusters.

To establish the effect of GDEC clustering we compared the chromosomal composition of clusters derived from GDEC and standard flexible beta clustering, using ER+ samples from three published breast cancer gene expression datasets [11,16-19]. The results indicate a clear enrichment in clusters with a high proportion of genes from the same chromosome in the GDEC clustered data (green versus red lines in Figure 1). These results were also compared to those obtained when the correlation structure in the datasets was destroyed by permuting the values in each row (gene) in the data matrix. The enrichment profiles for the permuted data are only slightly higher than that expected when chromosomes are randomly assigned to clusters (Figure 1). At the parameter settings used in this study, the influence of genomic distance does not dominate the gene-expression data, but rather reveals genomic regions of correlated gene expression. Thus, GDEC clustering gives a significant enrichment in genes from the same genomic locus in individual clusters, compared to traditional clustering techniques.

### Identification of recurrent regional metagenes

**Figure 2:**
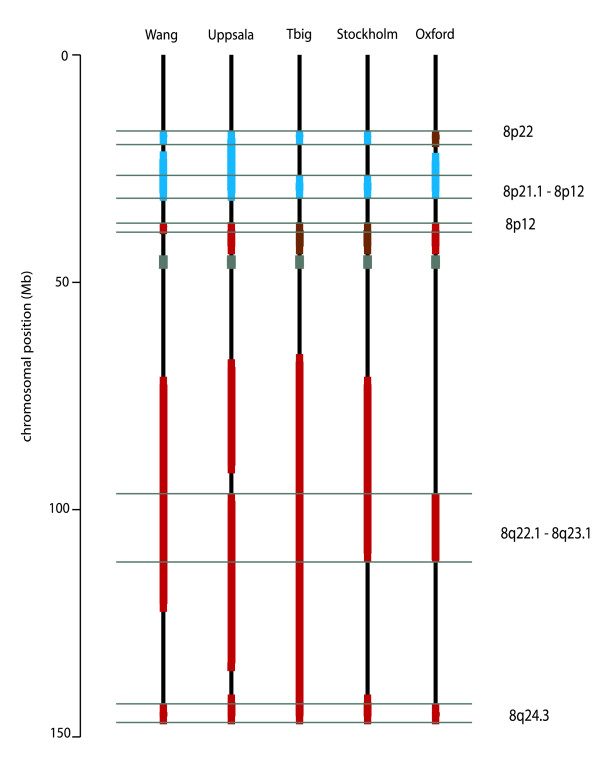
**Common regions of co-expression on chromosome 8**. The extent of the regional clusters for chromosome 8 are shown as coloured bars for each of the five datasets used. The regions of overlap between the studies are delineated by horizontal lines with the cytoband ranges labelled on the right. Although not indicated, the Tbig dataset has two overlapping regional metagenes on the q-arm.

A tree-cutting method was used to segregate clusters from the dendrogram generated by GDEC clustering. Regional metagenes (RMGs) were then defined as the centroids of clusters containing >90% genes from the same chromosome, giving rise to between 31 and 45 clusters per dataset. Comparison of the metagenes across studies revealed a number of regions conserved in at least four out of five datasets (Additional File 1), suggesting that this analysis has identified recurrent biological features. Permutation analysis yielded a maximum intersection gene list size of 10 genes only 3 times in 100,000 trials inferring a p-value close to zero for the 234 genes found in common to the regional metagenes listed in Additional File 1. Among the recurrently identified regions, differences were found in the extent of these clusters, as illustrated in Figure 2, which indicates the overlaps in the regional clusters on chromosome 8 for the five datasets. A marked sample set dependency was observed for many regions represented in less than four out of the five datasets.

### Regional metagenes, copy number aberrations and proliferation

The prognostic significance of the proliferative phenotype in these tumours as assessed by the proliferation signature has been emphasized by others [7,10]. In order to determine the relationship between RMGs and proliferation, we derived a proliferation metagene. For this we identified the cluster containing most of the genes reported in published proliferation signatures [7-9] in each of the three larger datasets then defined the proliferation metagene as the intersection of these three clusters (see methods for further details). Histograms of correlations of the most variable genes to the proliferation metagene are given in Additional File 2, and the genes that comprise the proliferation signature are detailed in Additional File 3. In each dataset the genes that constitute the proliferation cluster form a small shoulder in the distribution with correlations greater than 0.5. In order to use the proliferation metagene as a continuous marker of proliferation, we excluded all genes with a high correlation to the proliferation metagene (correlation >0.5) from the analysis prior to clustering. We demonstrate in a subsequent section that the proliferation metagene is a reliable surrogate of tumour grade [10,12] and results in a good separation of grade 1 and 3 tumours (Figure 4).

**Figure 3:**
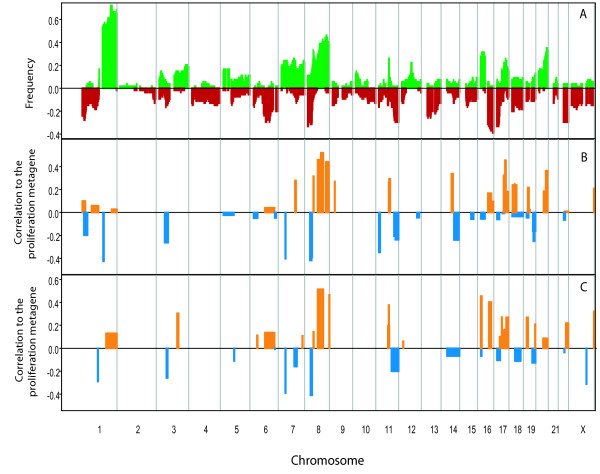
**Comparison of regional metagenes and DNA copy number changes**. A) Frequency plot of DNA copy number changes from 43 ER+ breast tumour CGH profiles [20]. B) Corresponding map of regional metagenes from matching gene expression data. C) Regional metagenes from the merged dataset of 793 tumours. In B) and C) the height and direction of the plotted peaks indicate the correlation of the RMGs to the proliferation metagene from the corresponding datasets.

To examine the possible correspondence between regional metagenes, DNA copy number changes and proliferation we used a study detailing parallel gene expression and CGH copy number analysis for 43 ER+ breast tumours [20]. We constructed a frequency plot for copy number aberrations (CNAs) from the CGH data and plotted this in parallel with the correlation of the RMGs to the proliferation metagene from the paired gene expression data in this study (Figure 3). Recurrent RMGs on chromosomes 3, 8, 11, 17 and 20 exhibit a pattern in which most copy number gains and losses correspond to positive and negative correlation to the proliferation metagene respectively. However, not all differentially expressed regions showed corresponding differences in DNA copy number (e.g. 7p15) suggesting that alternative mechanisms of gene expression regulation, such as epigenetic repression, are important in defining regional metagenes. DNA copy number loss and epigenetic silencing may also be alternative or additive mechanisms at regions such as 3p21 [21]. This demonstrates the integrative power of our approach, and its potential to define areas where copy number changes have real phenotypic consequences.

**Figure 4:**
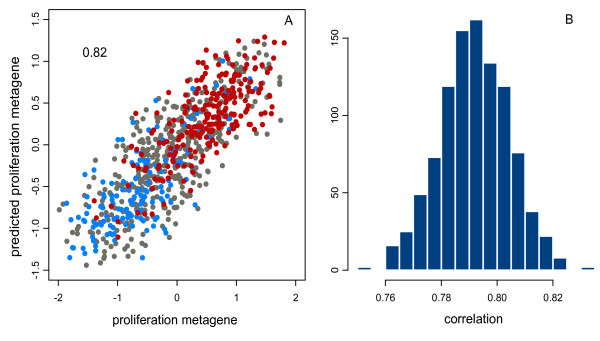
**Prediction of proliferation signal in combined dataset**. A) A scatter plot of showing the correlation to the proliferation metagene for the combined dataset containing 793 ER+ breast cancer samples, with the histological grade colour coded where blue is grade 1, red is grade 3 and grey indicates either grade 2 or missing value. The correlation of this fit is indicated in the upper left corner. B) A histogram of correlations for the predictions for 500 permutations in which the combined dataset was randomly split into two and each half was used to predict the proliferation metagene of the other half. The lambda value was fixed at an optimal value of 120 based on a prior set of cross-validations.

### Identification of regional metagenes predictive of proliferation

To investigate if regional clusters of co-expressed genes are predictive of proliferation, ridge regression was used to calculate weightings for regional metagenes when the proliferation metagene was used as the response variable (see methods). To validate the method we tested the regression models derived from individual datasets by using the other four datasets as validation datasets. In each case, RMGs were extracted from the validation datasets exactly as for the training dataset and the predicted proliferation metagene was calculated using the weightings derived from the training dataset. In most cases the correlations for the training sets were higher than the test predictions, indicating some degree of overfitting (Table 1). However, the test-set correlations were all significant, suggesting that the predictive significance of the RMGs and their calculated regression weightings were transferable between datasets. Thus, despite sample set differences in the RMGs, there was a repeated pattern of relationships between the proliferation metagene and the regional clusters of co-expressed genes.

**Table 1:** Correlation of the RMG regression fit across datasets

	Oxford	Stockholm	Tbig	Uppsala	Wang
Oxford	0.80	0.60	0.69	0.62	0.65
Stockholm	0.80	0.84	0.79	0.78	0.65
Tbig	0.73	0.68	0.82	0.66	0.62
Uppsala	0.76	0.74	0.75	0.82	0.76
Wang	0.67	0.63	0.66	0.69	0.78

The rows of this table represent the test datasets used and the columns represent the training datasets used to derive the RMGs and determine their regression weightings. The leading diagonal represents the correlations of the training models.

### Regional metagenes contribute additively to proliferation

To simplify the analysis and increase the sample size, five datasets were merged (see methods). We used GDEC clustering and the tree cutting method to derive 42 RMGs, and performed regression analysis as above (Additional File 4). The training fit gave a correlation of 0.82 to the proliferation metagene. To estimate the extent of overfitting we randomly split the dataset into two halves, and used both as a training set to derive weightings to predict the proliferation metagene of the other. This was repeated 500 times giving an average correlation of 0.79. Thus, by using a larger dataset we have reduced overfitting (see Figure 4).

A key question for this analysis is whether the multivariate regression models used here significantly improve on the correlation of the best individual RMG in the set of 42. The RMG with the highest absolute correlation to the proliferation signature was that at chromosome 8q13-8q22, with a correlation of 0.52 (Table 2). The lowest of the permutation test correlations was 0.75 (Figure 4b), and this conservative estimate of the accuracy of the model was still significantly higher than the most informative RMG (Fisher's z-score method, p-value 1.35^E-8^). We conclude that the RMGs provide information additively in predicting proliferation. As the most informative RMGs reflect common CNA's or regions of epigenetic silencing, we suggest that these genomic modifications act co-operatively and additively to produce cancers with higher proliferative capacity.

**Table 2:** Selection order for regional metagenes in prediction of proliferation

RMG	Chr	Cytobands	Position (Mb)	Genes	Correlation
1	8	8q13.1 - 8q22.3	66.72 - 104.15	21	0.52
2	8	8p22 - 8p12	17.55 - 31.15	19	-0.41
3	11	11q13.1 - 11q13.4	66.01 - 70.89	11	0.38
4	7	7p15.2 - 7p15.2	27.15 - 27.18	4	-0.39
5	3	3p21.31 - 3p14.3	49.13 - 58.5	9	-0.26
6	16	16p13.3 - 16p13.2	0.04 - 8.86	12	0.45
7	17	17p13.3 - 17p11.2	0.59 - 19.71	19	-0.11
8	22	22q11.22 - 22q11.22	20.88 - 21.57	5	-0.04
9	1	1q24.2 - 1q44	166.15 - 245	23	0.13
10	23	23q28 - 23q28	148.67 - 153.54	8	0.32
11	19	19q13.11 - 19q13.43	40.22 - 63.77	34	-0.13
12	3	3q13.32 - 3q22.1	120.41 - 132.22	8	0.30
13	1	1p13.3 - 1p13.3	110 - 110.09	4	-0.29
14	16	16p13.3 - 16p13.2	0.71 - 8.78	8	-0.07
15	16	16q13 - 16q22.3	55.32 - 73.24	16	0.40
16	22	22q12.2 - 22q13.33	28.46 - 49.31	20	0.22
17	23	23q22.1 - 23q22.2	99.77 - 102.52	6	-0.31
18	6	6q14.2 - 6q23.3	83.93 - 137.41	9	0.14
19	7	7q21.12 - 7q22.3	86.81 - 107.05	9	-0.16
20	20	20p11.21 - 20q13.33	25.18 - 62.13	18	0.09
21	7	7q34 - 7q34	142.14 - 142.18	4	0.11
22	17	17p11.2 - 17q21.32	19.38 - 43.11	15	0.10
23	11	11q14.1 - 11q25	85.05 - 133.6	13	-0.20
24	11	11q12.2 - 11q13.4	60.86 - 72.62	11	0.20
25	5	5q13.1 - 5q13.2	69.21 - 70.46	8	-0.11
26	8	8q24.3 - 8q24.3	141.6 - 146.25	19	0.47
27	11	11q22.3 - 11q24.3	109.61 - 129.59	11	-0.02
28	17	17q23.3 - 17q25.3	58.86 - 78.25	24	0.27
29	18	18q11.2 - 18q21.33	17.48 - 59.14	14	-0.11
30	17	17q21.31 - 17q24.1	38.06 - 59.92	17	0.16
31	22	22q12.3 - 22q13.1	34.37 - 37.81	10	0.08
32	12	12p13.31 - 12p13.2	6.42 - 10.48	10	0.06
33	17	17q11.2 - 17q21.31	23.39 - 39.99	19	0.08
34	17	17q22 - 17q24.2	53.3 - 64.04	9	0.16
35	17	17q12 - 17q21.1	33.04 - 35.61	8	0.27
36	19	19q13.42 - 19q13.42	59.41 - 59.84	4	0.21
37	6	6q25.1 - 6q25.1	151.77 - 152.46	4	-0.01
38	8	8p12 - 8p11.21	37.74 - 42.87	8	0.14
39	19	19p13.3 - 19p13.13	0.94 - 12.93	10	0.27
40	19	19q13.2 - 19q13.2	46.07 - 46.29	4	-0.06
41	14	14q32.33 - 14q32.33	105.4 - 106.35	4	-0.07
42	6	6p22.1 - 6p21.32	26.47 - 32.94	12	0.11

The RMGs are numbered in selection order. The chromosome, cytobands, position, number of genes and the correlation to the proliferation metagene for the merged 793- sample dataset are shown.

### Competitive selection of regional metagenes

**Figure 5:**
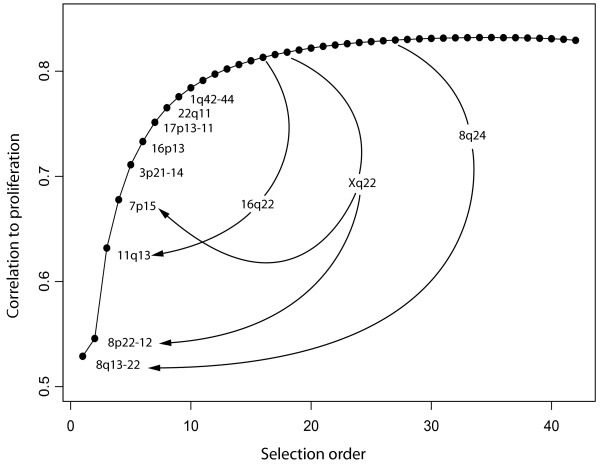
**The effect of RMG omission on selection order**. This graph plots the cumulative correlation to the proliferation metagene for the 42 RMGs used in the competitive selection analysis. The slope of the curve indicates that most of the correlation is explained by the top 13 RMGs. Omission of each of the top four RMGs in turn resulted in their replacement in the selection order with previously lowly selected metagenes (indicated by arrows).

To investigate the relative importance of RMGs in predicting proliferation we used a forward selection method in combination with sample subset permutations. A hundred random samples, each of 396 tumours, were drawn from the merged dataset of 793 profiles. For each sample, the RMG with the highest absolute correlation to the proliferation metagene was chosen as the seed RMG. The RMG that best improved the regression fit was then selected from those remaining and added to the model, until all 42 RMGs were included. The selection order was recorded for each of the 100 permutations and the ranks were averaged to give the final selection order (Table 2). The cumulative correlation of the fit to the proliferation metagene at each step is depicted in Figure 5.

Since the majority of the correlation is explained by the first few metagenes added to the model, we ran the selection order permutations four further times with each of the first four RMGs omitted of the RMG set, in order to observe the rank order changes that resulted. The arrows in Figure 5 indicate the metagenes that most frequently substituted for the omitted RMG. The substituting RMGs that replaced the deleted RMGs were not surprisingly correlated with them, illustrating some redundancy amongst the RMGs. In the first case the RMG at 8q24 is frequently gained along with the region at 8q13-22 and is highly correlated to proliferation, but was pushed down the selection order presumably because it provided redundant information once the 8q13-22 RMG had been selected. This effect caused the selection order to deviate from a decreasing order of absolute correlations. For example, the third RMG selected, 11q13 has a lower correlation to the proliferation signature (0.38) than the RMG at 8q24 (0.47). In selection order analyses for the individual datasets we consistently found that the top two RMGs contained an RMG at 8p22 together with one of three RMGs from 8q (data not shown). The RMGs on 8q probably carry redundant information and possibility reflect the common gains of the q-arm of chromosome 8 in breast cancer [20]. Consequently, the 11q13 RMG was more consistently able to provide additional, non redundant information to the model than a second RMG from 8q. Thus, our method establishes not only the regions that contribute most to proliferation, but also highlights the relationships between them such that the more orthogonal, and consequently the most additive combinations are selected with higher priority.

### Model Validation

The top three RMGs that were selected using our method reflect known genomic copy number aberrations in breast cancer, thus validating this method. Furthermore, the positively correlated RMGs 8q13-22 and 11q13 are in regions known to be gained, and the negatively correlated 8p22 RMG in a region of common loss [20]. Indeed, comparisons of DNA copy number changes between luminal A and luminal B type breast cancers, corresponding to low and high grade respectively, indicated that the frequency of gain on 8q and loss on 8p was much greater in the more proliferative luminal B subtype [20].

This analysis approach also highlighted the importance of the HOXA cluster at 7p15 (RMG4), which has been shown to undergo epigenetic silencing in tumours [22]. The HOXA genes have been implicated in growth suppression and apoptosis via a p53-dependent pathway [23,24]. The consistent selection of the HOXA cluster on chromosome 7p15 in the sequential model building method, suggests that down-regulation of these genes is an additive event and not simply a consequence of rearrangements on chromosome 8.

The metagene at 3p21-14 (RMG5) contains the gene IL17BR. This gene forms half of a two gene predictor for response to tamoxifen treatment, along with HOXB13. IL17BR has been shown to be significantly negatively correlated to grade at the expression level in a large panel of tumours [25], and is in a region where loss has been associated with high grade [1].

Chromosome 1 frequently exhibits gain of the q arm and loss of the p arm in breast cancer [20], with loss of the p arm more frequent in luminal B type tumours. We identified a region from 1q24-44 (RMG9) that was positively correlated to proliferation, and a region at 1p13 (RMG13) that was negatively correlated to proliferation. Thus, this analysis can help pinpoint the location of genes that drive cancer progression when amplified or lost.

The metagene at 17p13 (RMG7) sits in a region that undergoes copy number loss more frequently in luminal B compared to luminal A tumours [20]. This RMG spans the p53 gene and was negatively correlated to proliferation. Epigenetic silencing at 7p15 and copy number loss at 17p13 can both affect progression of tumours with wild type p53, but are likely to be less important in tumours that harbour p53 null mutations.

### Detailed analysis of regional metagene interactions

**Figure 6:**
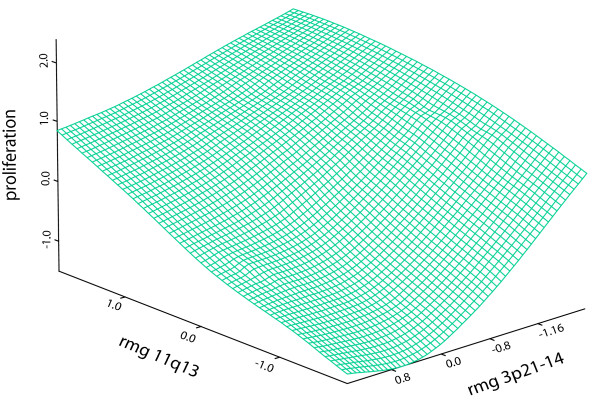
**Additivity of two RMGs in predicting proliferation**. This surface plot depicts additivity between the third (11q13) and fifth (3p21-14) RMGs in the prediction of the proliferation metagene. The plot was generated using locally weighted robust regression (Loess) at a span of 0.75.

An advantage of our method is that it can provide information about genes not originally included in the analysis by indicating the relative importance of a region. The fifth most informative RMG at 3p21 spans the RASSF1 tumour suppressor gene. Although this gene was not included in this study, the expression data for this gene was found to be negatively correlated with the proliferation metagene and positively correlated with the metagene at 3p21 (data not shown). This tumour suppressor gene is frequently subject to epigenetic silencing via a mechanism involving the regional spreading of heterochromatin following failure of CTCF dependent insulator sites [21]. In addition, RASSF1 negatively regulates the accumulation of cyclin D1 at a post-transcriptional level [26], suggesting a potential example of an additive interaction, between cyclin D1 containing RMG at 11q13 and the region at 3p21 (Figure 6). Bioinformatic data mining of interactions and annotations of genes within interacting genomic regions can be used to generate hypothesis for functional studies to identify the key genetic interaction within differentially expressed regions.

### Interaction network analysis of regional metagenes

**Figure 7:**
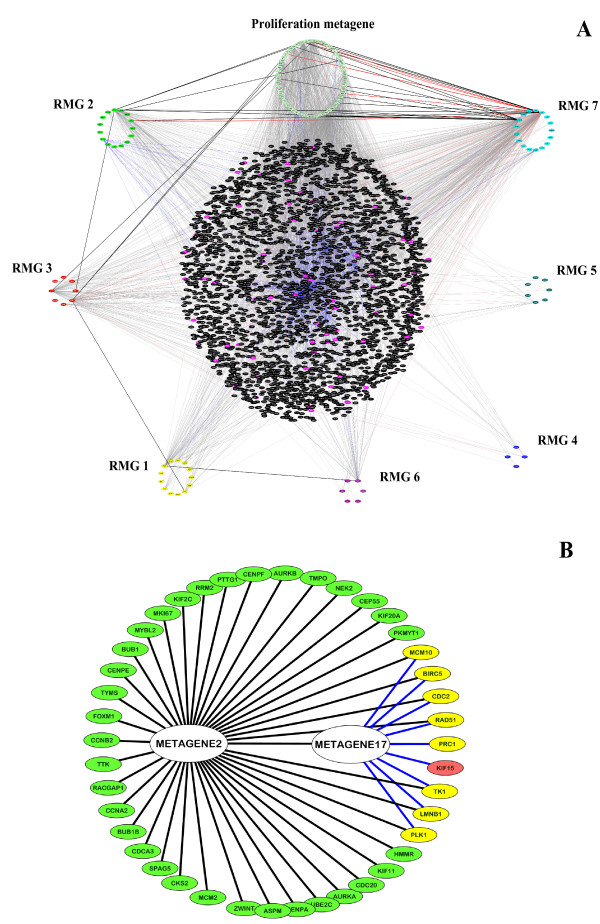
**Interaction network associations of regional metagenes**. A) Overview of the interaction network defined by the proliferation metagenes and RMGs 1 to 7 (8q13-22, 8p12-22, 11q13, 7p15, 3p14-21, 16p13, 17p11-13 respectively). Light gray edges, protein-protein interactions, red lines, transcriptional interactions, blue lines, interactions within protein complexes. Genes from RMGs 8 to 42 participating in signalling within the network core are shown in magenta. Direct interactions between the proliferation metagenes and top seven RMGs gene members are emphasized in bold. B) Close association in proliferation metagenes gene targets between RMG 2 (8p12-22) and RMG 17 (23q22). Proliferation metagenes genes targeted only by RMG 2 are shown in green, those targeted only by RMG 17 are shown in salmon pink and those targeted by both RMGs are highlighted in yellow.

We have observed that most of the variation of the proliferation metagene can be explained by the first seven regional metagenes, and that these metagenes can be replaced by others initially much further down the selection order. We hypothesised that RMGs at the top of the selection order should carry non-redundant information, and thus be involved in distinct pathways related to common cell-biological processes. This led us to investigate the networks of characterised protein interactions between these top seven RMGs and the proliferation metagene (Figure 7A and Additional File 5).

In most cases connections between RMGs were largely mediated through a centralised component (shown in black) comprising 2808 genes (36 belonging to RMGs 8 to 42) and 3050 interactions, the majority of which emanated from a small number of non-metagene hubs (BRAF, RAF1, DDB1, IMMT, DLG4, TRAF6 and HCLS1). Most of these hubs interacted directly with genes in the proliferation cluster. In the case of RMG 7 a significant number of direct interactions with the proliferation metagene were observed, with TP53 and PAFAH1B1 interacting with 17 proliferation metagenes components including CDC2, CCNA2, RAD51 and AURKA (Additional File 5).

To investigate the relationship between the top RMGs and those that replaced them when they were omitted, a protein interaction network was generated from the proliferation metagene and RMGs 1 to 4, 15, 17 and 26 (8q13-22, 8p12-22, 11q13, 7p15, 16q13-22, 23q22 and 8q24 respectively) and the direct or shortest indirect signalling interactions with the proliferation metagene members were compared. For the top 3 RMGs (8q13-22, 8p12-22 and 11q13), the replacing metagene hit a subset of the respective proliferation metagene members, indicating some functional equivalence (Figure 7B and Additional File 6). Interestingly, this overlap was not observed for RMG 4 (7p15) suggesting that, for the HOX cluster, signalling to the proliferation metagene may be mediated through additional interactions within the centralised component (Figure 7A shown in black).

This analysis reiterates the finding that a number of small changes in a set of complementary pathways driving cell growth and division can act additively to increase cell proliferation. Furthermore, analysis of the RMGs that carry redundant information can help to narrow down the list of potential cancer drivers within RMGs.

## Conclusions

We have shown that a small regional distortion of correlation distance in agglomerative clustering results in the formation of regional clusters of co-regulated genes. We have constructed metagenes from these clusters and used linear regression in the modelling of grade using proliferation as a surrogate. Using this approach we have identified 42 genomic regions where gene expression is recurrently altered in ER+ breast cancers. We have gone on to identify the regions most correlated with the proliferation signature. We show that distinct genomic regions combine additively to enhance proliferation, and that regions can be ranked by their contribution to the proliferation rate in a competitive model. As a result we have identified the differentially regulated genomic regions that are most important in proliferation, and hence grade and prognosis, of ER+ breast cancer. Furthermore, detailed analysis of the interacting regions has identified a number of possible genetic drivers of cancer that are involved in key cellular pathways. This approach will have utility in identifying and integrating chromosomal regions where coordinate changes in gene expression confer clinically relevant cancer phenotypes.

## Methods

### Microarray data normalisation

Microarray gene expression data for five of the breast cancer datasets used in this study, were obtained from the GEO database (GSE6532, GSE1456, GSE3494, GSE7390, GSE2034) [27]. The paired gene expression and array comparative genomic hybridization data for 43 ER+ tumours [20] was downloaded from the database referenced therein. The gene expression data from all these studies was derived using the Affymetrix 133A platform, comprised of the 22215 non-control probesets. ER+ tumour samples were selected on the basis of histological sample annotation. MAS5 processed gene expression values were log2 transformed, and then quantile normalization was applied across all samples [28]. Following transformation and normalisation probesets that had a maximum expression level below 7, an inter-quartile range below 0.5, or missing genomic mapping information were excluded from the analysis. The gene expression values for each gene were then standardised to a mean of 0 and standard deviation of 1 within each dataset. In the case of the combined dataset, this resulted in a final matrix of 5466 probesets for 793 tumours.

The normalized aCGH data from [20] was smoothed using the DNAcopy R package from Bioconductor [29]. Gains and losses were defined as those log2 ratio values that exceeded 0.2 and were less than -0.2 respectively.

### Genomic distance entrained clustering algorithm

The GDEC clustering method used a modification of the gene-gene correlation distance matrix prior to clustering by the flexible beta agglomerative clustering algorithm ($\frac{1-\beta }{2}$, -1 <*β *< 1) with *β *= -0.5, because for small negative values of this parameter the space dilation during clustering avoids chaining in the dense gene space [30]. For genes on the same chromosome a sigmoid function was used to control the influence of genomic distance;

$$g_{ij}=1-\frac{1}{1+\exp(-a(x_{ij}-h))},$$

where *x_ij _*is the absolute genomic distance between genes *i *and *j *in megabases, *h *is a parameter setting the distance of half maximum influence, and *a *is parameter controlling the steepness of change in influence with distance. The genomic distance adjusted correlation distance, *d_ij _*between genes *i *and *j*, is then calculated by the following function;

$$d_{ij}=\lambda g_{ij}\frac{2}{1+\exp(2\pi \rho_{ij})}+(1-\lambda g_{ij})(1-\rho_{ij})$$

where *ρ_ij _*is the Pearson correlation, and *λ *is a scaling parameter that controls the extend of distance distortion. In this study the parameters where fixed at; *a *= 0.25, *λ *= 0.5, *h *= 10 Mb. A three-dimensional plot of the function is provided in additional file 7.

### Metagene construction

The proliferation metagene was derived as follows. The most variable genes from the three larger datasets used here (Tbig, Uppsala and Wang) were clustered by standard flexible beta clustering and the dendrograms cut to give 100 clusters. In each of the three sets of 100 clusters, a single cluster was identified that contained most of the genes found in published proliferation signatures [7-9]. The gene list used to derive the proliferation metagene used here was taken as the intersection of these three clusters. When the proliferation metagenes were derived *de novo *for each of the individual datasets, by identifying the proliferation cluster as above, the correlation of this *de novo *proliferation metagene to the proliferation metagene from the intersection gene list was always high (worst case 0.956). This supports the use of metagenes as stable and transferable estimates of recurrent expression patterns.

All metagenes were derived as the mean vector of the genes in the corresponding cluster following standardization to mean of 0 and standard deviation of 1. In order to avoid biased gene weighting, values from duplicate probesets for the same gene (UniGene Cluster) were averaged prior to averaging across different genes in metagene calculation. For clusters that derived less than 100% of their genes from the same chromosomal region (i.e. 90% to100%), the minority genes from different regions were excluded from the calculations.

### Ridge regression and competitive selection

Unless otherwise stated, ridge regression was used with the ridge parameter set by leave-one-out cross-validation in the training set (values ranged from 25 to 120).

Competitive selection was carried out on the merged dataset of 793 ER+ samples from the GSE6532, GSE1456, GSE3494, GSE7390 and GSE2034 datasets. One hundred random sample sets, each with 396 tumours, were drawn from the pool. The ridge regression model was then built up selecting the RMG at each step that best improved the fit. The ridge parameter was fixed at a value of 25 for this analysis. The average RMG rank and correlation of the fit at each step was then recorded.

The clustering and derivation of RMGs was not repeated for each permutation in this analysis, and thus we are only testing the consistency of the regression weightings given a fixed set of RMGs.

### Interaction network analysis

Gene lists from the proliferation metagene and RMGs 1 to 7 (and subsequently RMGs 15, 17 and 26) were submitted to ROCK [31] for network generation http://rock.icr.ac.uk. The resultant network was visualised with ROCKscape (manuscript in preparation), a modification of Cytoscape http://www.cytoscape.org that allows integration with ROCK-BCGF. Network metrics were derived with the RandomNetworks plugin http://sites.google.com/site/randomnetworkplugin/.

## Authors' contributions

TD conceived the study, programmed the algorithms and carried out the statistical analyses. DS assisted in study design and DS drafted the manuscript. CM performed the network analysis. AM and AG retrieved and annotated gene expression datasets. AA edited mathematical equations (Latex) for publication. All authors read and approved the final manuscript.

## Supplementary Material

Additional file 1 **Genomic regions co-expressed in four out of five datasets**. This excel spreadsheet lists the Genomic regions co-expressed in 4/5 datasets. Regions are given in megabases. Cluster refers to a cluster of paralogs from the same gene family. Click here for file

Additional file 2 **Correlation of variable genes to proliferation**. Histograms of correlations of gene expression to the proliferation metagene for the 5466 most variable genes from ER+ tumours in five datasets. The cluster of genes that constitute the proliferation metagene form a small peak or shoulder at high correlation. Click here for file

Additional file 3 **The microarray probes included in the proliferation signature**. This excel spreadsheet lists the microarray probes from the Affymetrix U133A platform that were included in the proliferation metagene in this study, along with their gene annotations. Click here for file

Additional file 4 **The microarray probes included in the 42 regional metagenes**. This excel spreadsheet lists the microarray probes from the Affymetrix U133A platform that were included in the 42 regional metagene in this study, along with their gene annotations. Click here for file

Additional file 5 **The interaction network defined by the proliferation metagenes and the top seven regional metagenes**. The cytoscape file used to generate Figure 7A. Click here for file

Additional file 6 **Proliferation metagene targets hit by regional metagenes**. This Word document describes the proliferation metagene genes targeted by (A) RMG 1 and RMG 26 (8q13-22 and 8q24) (B) RMG 2 and RMG 17 (8p12-22 and Xq22) (C) RMG 3 and RMG 15 (11q13 and 16q13-22) and (D) RMG 4 and RMG 17 (7p15 and Xq22). See Figure 7B legend for details. Click here for file

Additional file 7 **Distance function for GDEC clustering**. The GDEC clustering method uses a local distortion of the correlation distance between genes in the same chromosomal region. The three dimensional plot illustrates the function used to relate genomic distance and correlation to the output distance. The red line indicates the unadjusted correlation distance at a genomic distance of zero. Click here for file
