# Supplementary material for: Genomic distance entrained clustering and regression modelling highlights interacting genomic regions contributing to proliferation in breast cancer
Source: BMC Syst Biol. 2010 Sep 8;4:127. doi: 10.1186/1752-0509-4-127 (PMC2946304; doi:10.1186/1752-0509-4-127)
Supplement: Additional file 2 — Correlation of variable genes to proliferation. Histograms of correlations of gene expression to the proliferation metagene for the 5466 most variable genes from ER+ tumours in five datasets. The cluster of genes that constitute the proliferation metagene form a small peak or shoulder at high correlation. [file 1752-0509-4-127-S2.DOC]

**Correlation of variable genes to proliferation**
