# Supplementary material for: Genomic distance entrained clustering and regression modelling highlights interacting genomic regions contributing to proliferation in breast cancer
Source: BMC Syst Biol. 2010 Sep 8;4:127. doi: 10.1186/1752-0509-4-127 (PMC2946304; doi:10.1186/1752-0509-4-127)

# Additional File 8- Proliferation metagene targets hit by regional metagenes

This Word document describes the proliferation metagene genes targeted by (A) RMG 1 and RMG 26 (8q13-22 and 8q24) (B) RMG 2 and RMG 17 (8p12-22 and Xq22) (C) RMG 3 and RMG 15 (11q13 and 16q13-22) and (D) RMG 4 and RMG 17 (7p15 and Xq22). See Figure 6B legend (main text) for details.

(A)


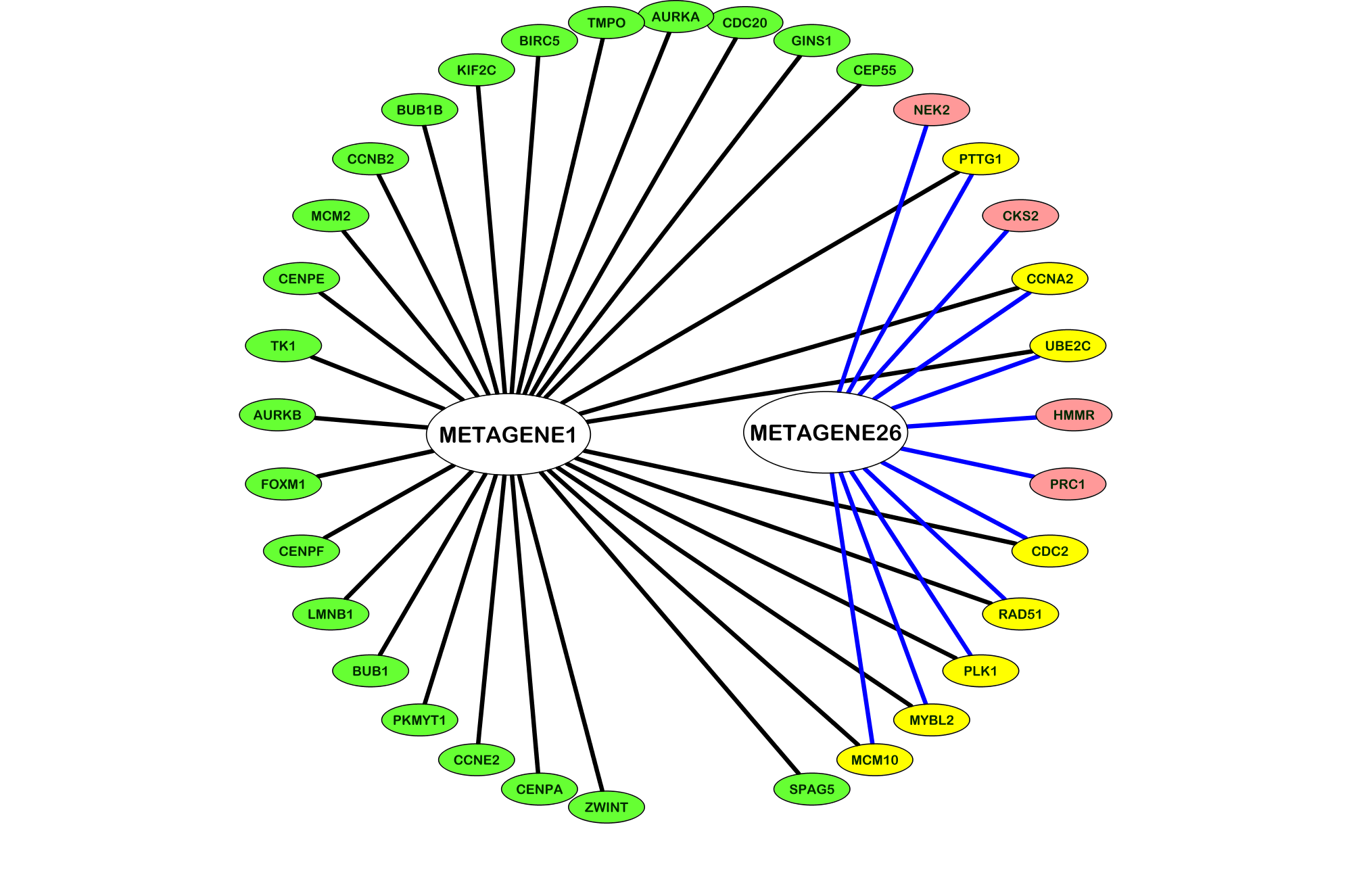


(B)


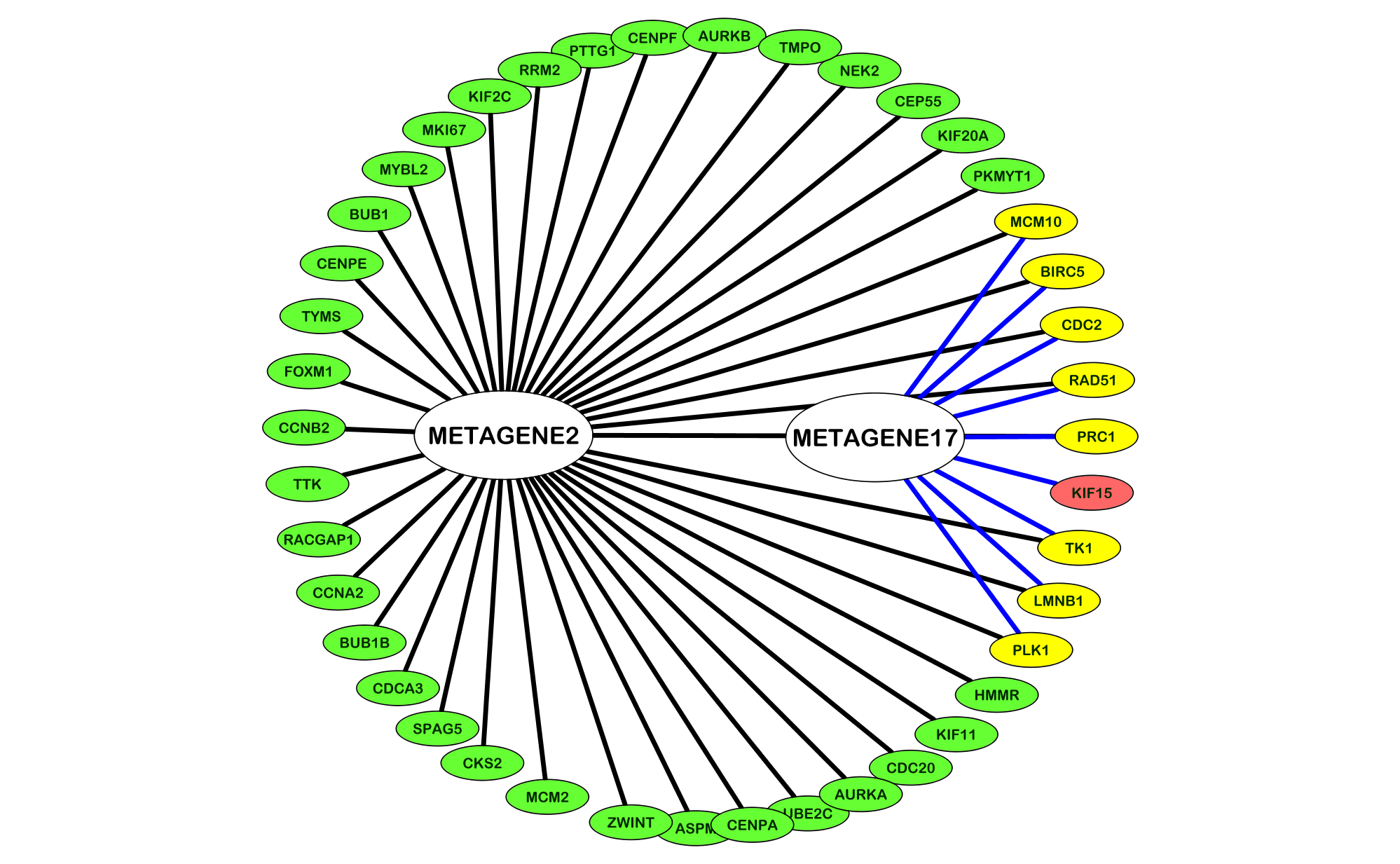


(C)


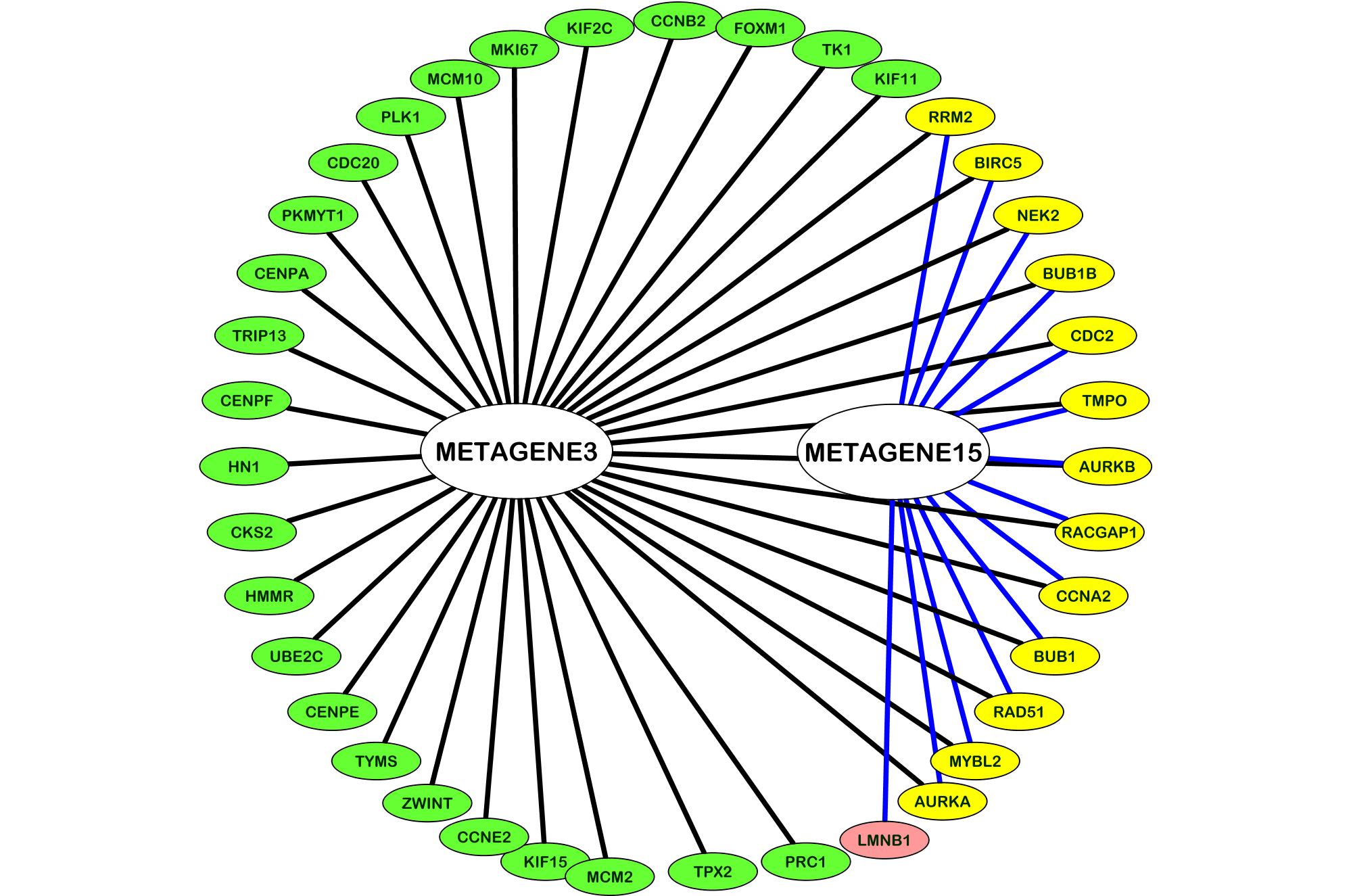


(D)


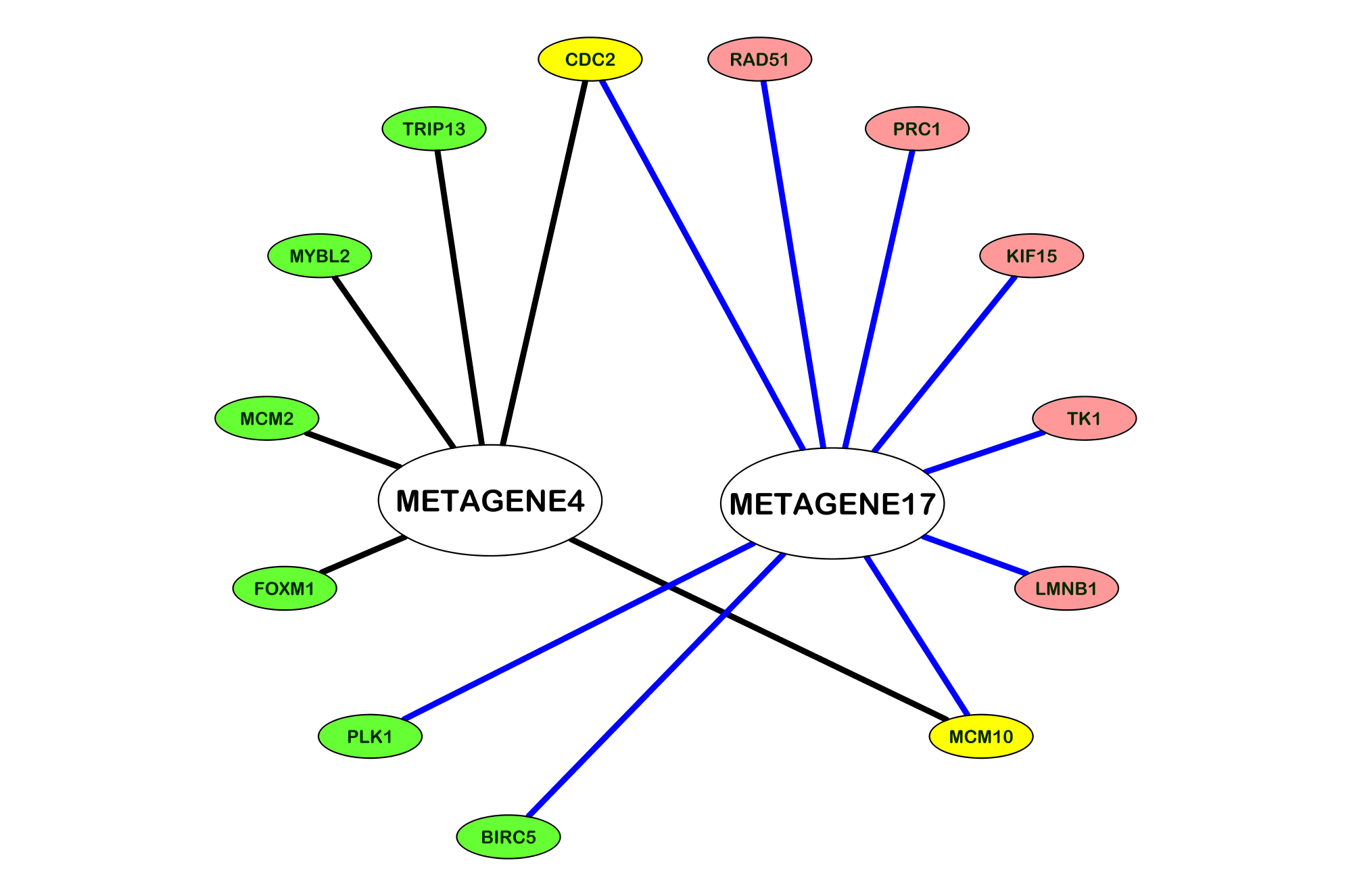

Supplement: Additional file 6 — Proliferation metagene targets hit by regional metagenes. This Word document describes the proliferation metagene genes targeted by (A) RMG 1 and RMG 26 (8q13-22 and 8q24) (B) RMG 2 and RMG 17 (8p12-22 and Xq22) (C) RMG 3 and RMG 15 (11q13 and 16q13-22) and (D) RMG 4 and RMG 17 (7p15 and Xq22). See Figure 7B legend for details. [file 1752-0509-4-127-S6.DOC]
