# Supplementary figures and images for: Genomic distance entrained clustering and regression modelling highlights interacting genomic regions contributing to proliferation in breast cancer
Source: BMC Syst Biol. 2010 Sep 8;4:127. doi: 10.1186/1752-0509-4-127 (PMC2946304; doi:10.1186/1752-0509-4-127)

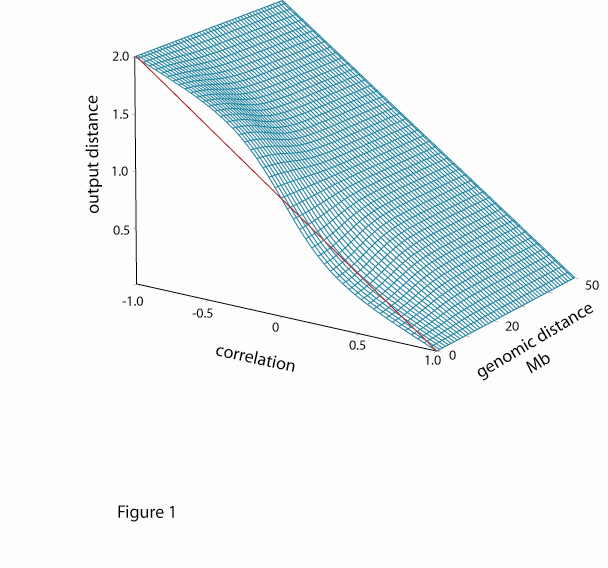


## Additional File 1 - Distance function for GEDI clustering

Supplement: Additional file 7 — Distance function for GDEC clustering. The GDEC clustering method uses a local distortion of the correlation distance between genes in the same chromosomal region. The three dimensional plot illustrates the function used to relate genomic distance and correlation to the output distance. The red line indicates the unadjusted correlation distance at a genomic distance of zero. [file 1752-0509-4-127-S7.DOC]
